# Provider reported implementation barriers to hepatitis C elimination in Washington State

**DOI:** 10.1186/s12875-024-02507-0

**Published:** 2024-07-11

**Authors:** Paula Cox-North, Lisa Wiggins, Jon Stockton, Emalie Huriaux, Mary Fliss, Leta Evaskus, Kenneth Pike, Anirban Basu, Pamela Kohler

**Affiliations:** 1 Department of Biobehavioral Nursing and Health Informatics, University of Washington, Box 357260, Seattle, Washington, 98195 USA; 2 Department of Child, Family, and Population Health Nursing, University of Washington, Box 357260, Seattle, Washington, 98195 USA; 3 Department of Health, Washington State, Tumwater, Washington, USA; 4 Washington State Office of Financial Management, Olympia, WA USA; 5 Washington State Health Care Authority, Olympia, WA USA; 6 Office of Nursing Research, University of Washington, Box 357260, Seattle, Washington, 98195 USA; 7 Department of Pharmacy, University of Washington, Box 357630, Seattle, Washington, 98195 USA; 8 Department of Global Health, University of Washington, Box 351620, Seattle, Washington, 98195 USA

**Keywords:** Hepatitis C, Substance use, Stigma, Barriers, Washington State

## Abstract

**Background:**

Despite curative treatment options since 2014, only 12% of individuals in Washington State diagnosed with Hepatitis C (HCV) received treatment in 2018. Washington State agencies launched an elimination plan in 2019 to promote access to and delivery of HCV screening and treatment. The purpose of this study is to evaluate provider and health system barriers to successful implementation of HCV screening and treatment across Washington State.

**Methods:**

This is a cross-sectional online survey of 547 physicians, nurse practitioners, physician assistants, and clinical pharmacists who provide care to adult patients in Washington State conducted in 2022. Providers were eligible if they worked in a primary care, infectious disease, gastroenterology, or community health settings. Questions assessed HCV screening and treating practices, implementation barriers, provider knowledge, observed stigma, and willingness to co-manage HCV and substance use disorder. Chi-squared or fishers exact tests compared characteristics of those who did and did not screen or treat.

**Results:**

Provider adoption of screening for HCV was high across the state (96%), with minimal barriers identified. Fewer providers reported treating HCV themselves (28%); most (71%) referred their patients to another provider. Barriers identified by those not treating HCV included knowledge deficit (64%) and lack of organizational support (24%). The barrier most identified in those treating HCV was a lack of treating clinicians (18%). There were few (< 10%) reports of observed stigma in settings of HCV treatment. Most clinicians (95%) were willing to prescribe medication for substance use disorders to those that were using drugs including alcohol.

**Conclusion:**

Despite widespread screening efforts, there remain barriers to implementing HCV treatment in Washington State. Lack of treating clinicians and clinician knowledge deficit were the most frequently identified barriers to treating HCV. To achieve elimination of HCV by 2030, there is a need to grow and educate the clinician workforce treating HCV.

## Background

Hepatitis C (HCV) is a common bloodborne infection, with 56.8 million individuals affected globally, including an estimated 2.2 million individuals with chronic HCV infection in the United States [1]. Untreated chronic HCV is one of the leading causes of cirrhosis and liver cancer accounting for 290,000 deaths globally in 2019 [2], and has been associated with other co-morbid conditions such as cirrhosis and hepatocellular carcinoma [3]. The burden of HCV is especially high among younger individuals who inject drugs, which accounts for approximately 70% of new HCV infections in the United States [2], and a 60% increase of new HCV infections between 2017 and 2021 in Washington State [4].

More recently approved direct acting antivirals (DAAs) for HCV have demonstrated high rates of sustained viral response, good tolerability, and have shortened treatment duration to eight or twelve weeks of oral therapy. [5–9] Despite these advances in treatment, gaps persist in linkage to care and treatment completion. Of the 2 million infected individuals in the U.S., it is estimated that 40% are undiagnosed, and only 42% of those that are diagnosed had initiated treatment by the end of 2020 [1]. Washington State estimates, using projections developed by the Center for Disease Analysis Foundation, that only 12% of the 59,100 people with known active HCV infections in 2018 were connected to care and received HCV treatment [10].

To address the increase in infections in younger individuals, the Centers for Disease Control and Prevention (CDC), the US Preventive Services Task Force (USPTF), and the American Association for the Study of Liver Diseases–Infectious Diseases Society of America (AASLD–IDSA) have all endorsed universal screening for HCV in adults ≥ 18 years of age. [11–13] Globally, the World Health Organization Global Health Sector on viral Hepatitis 2016–2021 called for the global elimination of HCV by 2030, with a goal of treating 80% of those infected, reducing new infections by 90%, and reducing HCV related deaths by 65% [14].

In 2018, the Governor of Washington State announced a directive to eliminate HCV by 2030.

Washington’s statewide “Hep C Free Washington” plan was launched by the Healthcare Authority, Department of Health, and other stakeholder agencies in 2019, to develop recommendations for a comprehensive public health strategy [10]. Activities under the plan included enhancing public health HCV surveillance databases [15], securing a pricing agreement with the manufacturer of one of the antiviral medications, and implementation of different public health outreach and care integration strategies, aimed at improving access to marginalized populations, linking people to care, and building capacity of the healthcare workforce. Previously, through the Washington State Medicaid program, HCV treatment could only be offered through consultation with a specialist, but as part of this strategic plan, specialty consultation requirements were removed. In addition, by securing a pricing agreement, Medicaid was able to remove the prior authorization process and dispense an entire course at one time from any pharmacy covered by the patients plan. simplifying access to HCV medications [10].

Multiple barriers at patient, provider, and system levels have been identified globally to be barriers to HCV screening and treatment efforts. These include funding, high drug costs, provider treatment restrictions, lack of treatment access and services, gaps in medical knowledge, patient adherence, and stigma particularly in marginalized individuals and people who inject drugs [16–18]. To ensure that Washington State is on track to meet the elimination targets by 2030, a better understanding of provider barriers to HCV screening and treatment, especially in non-specialist settings, will help target the allocation of resources needed to bring these efforts to scale and help determine needs and pathways for optimal implementation. The purpose of this analysis is to describe current provider and health system factors that are barriers to larger scale implementation of HCV screening and treatment in Washington State.

## Methods

### Study design and population

This was a cross-sectional online survey study administered between March 10, 2022 and April 25, 2022. We emailed our survey along with a cover letter inviting participation of physicians, nurse practitioners, physician assistants, and clinical pharmacists who provide care to adult patients with HCV in Washington State, USA. We included clinical pharmacists in our survey because in Washington State pharmacists can prescribe and manage medications with a collaborative drug therapy agreement in place. The survey sample was derived from a list provided by the Washington State Department of Health public records center (*n* = 27,622). Providers were eligible to participate if they provided care for adults in primary care, infectious disease, gastroenterology, or community and public health care settings. Providers not in the requested care categories, or not interested in participation were given the option to ignore the survey or opt out of future reminders.

### Data collection

Survey design was guided by constructs from the Updated Consolidated Framework for Implementation (CFIR) and in consultation with Washington State health agency representatives, to assess provider practices and barriers to delivery of HCV screening and treatment in Washington State [19, 20]. Barrier measures were adapted from the literature [16–18], and stigma measures adapted from a brief stigma tool used in healthcare settings for those with HIV [21]. The survey consisted of 31 questions including provider role and clinic setting, HCV screening and treating practices, provider knowledge of current HCV practices, clinic or organization resources such as lab, phlebotomy, and staffing, stigma, and willingness to offer HCV treatment to those actively using drugs and/or alcohol [16–21]. We pilot tested surveys with a small sample of our target population prior to dissemination, and adjustments were made to refine the survey prior to full dissemination.

After the initial invitation was emailed to providers, three survey reminder emails were sent to those that had not responded to the survey and did not opt out over the six-week data collection period. Surveys were administered electronically using Research Electronic Data Capture (REDCap), which is a secure browser-based web application used for developing, maintaining, and managing different types of surveys and securing online data collection [22]. Once completed, survey responses were deidentified.

### Outcome and variable definitions

**Fig. 1:**
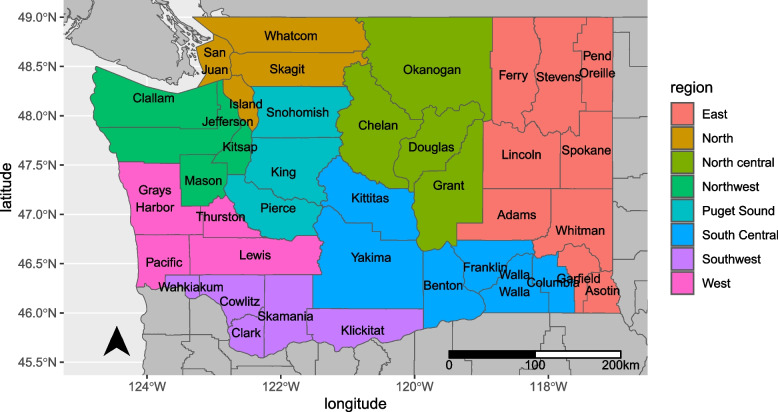
Geographic Regions of Washington State. Reference: US Census TIGER/Line data for 2021

Clinic setting was defined according to geographic region and facility type. Regions were reported by providers as county of practice and then consolidated in eight regions for analysis based on previous state initiatives [23], (Fig. 1). Facilities were categorized as; community and public health which are clinics dedicated to promoting, managing, and maintaining health in the populations they serve, private practice primary care, specialist, managed care which are coordinated health networks that provide services to members, and government or federal agency (department of corrections, veterans administration, and state inpatient mental health). Since participants could work at multiple sites they were asked to select all clinics/facilities where they worked, we grouped those reporting multiple organizations with those reporting “other” sites as the treatment practices may be different between the type of organization.

Primary outcomes were self-report of HCV screening (binary) and self-report of HCV treatment (treat, refer either within or external to my organization, or do not treat or refer).

### Analysis

We performed descriptive statistics for categorial variables using variable counts(n), and proportions (%). Comparisons of characteristics by screening and treatment approaches used Fisher’s exact or chi-squared tests, as appropriate with significance set at *p* ≤ 0.05. Analysis was conducted using Stata statistical software, version 18 (Stata Corp LLC) [24].

### Ethical considerations

This study received a non-research determination from the University of Washington Institutional Review Board, and a waiver of informed of consent was obtained.

## Results

### Demographics

Of the 27,622 licensed providers emailed, we received responses from 707 providers, among whom 137 did not answer any questions in the survey and 23 reported not meeting inclusion criteria, for a total of 547 participants. Demographic characteristics of participants are shown in (Table 1). Most respondents (66%) were physicians, (22%) were nurse practitioners, (11%) were physician assistants, and < 1% pharmacists. Just over half (51%) were from community health centers, which included hospital-based primary care outpatient clinics, and about one-quarter were based in private practice primary care clinics. Most providers (59%) practiced in the urban Puget Sound Region of Washington State, with (41%) of providers practicing in other more rural regions of the state.

**Table 1:** Demographics (*N* = 547)

Characteristic	n	%
**Provider type**		
Physician	362	66.2%
Nurse Practitioner	122	22.3%
Physician Assistant	59	10.8%
Pharm D*	4	0.7%
**Facility type**		
Community Health	279	51.0%
Public Health	23	4.2%
Private Practice Clinic	126	23.0%
Specialist Clinic	25	4.6%
Government/Federal Agency	25	4.6%
Managed Care Health Clinic	35	5.5%
Other/Multiple sites	30	2.9%
**Region of WA State**		
East	45	8.2%
North	34	6.2%
North central	18	3.3%
Northwest	31	5.7%
Puget Sound	321	58.8%
South Central	34	6.2%
Southwest	23	4.2%
West	40	7.3%

^*^Professional doctorate in pharmac*y*

### HCV screening

Almost all responding providers (96%) across the state reported screening for HCV (Table 2). There were significant differences in screening across clinic settings, with lowest rates in public health settings (78%), (*p* = 0.004). There were no differences in screening across providers and region. Screening tests are primarily being done by laboratory testing (70%) vs. point of care testing (5%), with over half of the laboratory testing (59%) being done by HCV antibody with reflex to PCR testing (performing an antibody test and an RNA test on the same specimen if the antibody test is reactive), reducing the need for multiple blood draws and tests to confirm HCV diagnosis.

**Table 2:** Screening by provider type, facility type, and region (*N* = 543)^a^

	**Do not screen** **(n = 24)**		**Screen** **(n = 519)**		
**Characteristic**	**n**	**%**	**n**	**%**	***p-value***
**Overall**	24	4.4	519	95.6	
**Provider Type**					0.174
Physician	14	3.9	348	96.1	
Nurse Practitioner	9	7.4	113	92.6	
Physician Assistant	1	1.7	58	98.3	
**Facility Type**					0.004
Community Health	7	5.6	271	97.5	
Public Health	5	21.7	18	78.3	
Private Practice Clinic	7	5.6	119	94.4	
Specialist Clinic	2	8.7	21	91.3	
Government/Federal Agency	0	0.0	25	100.0	
Managed Care Health Clinic	0	0.0	35	100.0	
Other/multiple sites	2	6.9	27	93.1	
**Region of WA State**					0.239
East	3	6.7	42	93.3	
North	0	0.0	34	100.0	
North central	0	0.0	18	100.0	
Northwest	0	0.0	31	100.0	
Puget Sound	13	4.1	305	95.9	
South Central	2	5.9	32	94.1	
Southwest	2	8.7	21	91.3	
West	4	10.3	35	89.7	

^a^Restricted to physician, nurse practitioner, and physician assistants since screening does not happen in pharmacy settings

### HCV treatment

When providers were asked about current treatment practices for HCV, fewer providers (28%) reported that they treated HCV themselves (Table 3). Most (71%) referred patients elsewhere for treatment and 1% reported that they do not treat or refer their patients. There were differences in treatment and referral practices by provider type (*p* = 0.002). All pharmacists (though only four in number) reported treating HCV. Approximately one third of physicians treated without referring, with slightly fewer nurse practitioners (19%) and physician’s assistants (24%). Specialists more frequently reported treating HCV than those in other clinical settings (*p* =  < 0.001). Private practice and managed health had the lowest proportion treating (15% and 17% respectively), though referral rates were high (85% and 83% respectively). Only those in community health and public health settings reported they did not treat or refer patients (1% and 13% respectively). There were no differences in treatment practices across the regions of the state. Among providers treating HCV, when asked about the number of patients treated in the last 2 years, most had treated 1–20 people (59%, 91/153).

**Table 3:** Treatment rate by provider type, clinic type, and region, (*N* = 544)^a^

	**Treatment approach**						
	**Treat**		**Refer**		**Neither Treat nor Refer**		*p-value*
**Characteristic**	**n**	**%**	**n**	**%**	**n**	**%**	
**Overall**^a^	153	28.1	384	70.6	7	1.3	
**Provider Type**							0.002
Physician	112	31.1	245	68.1	3	0.8	
Nurse Practitioner	23	19.0	94	77.7	4	3.3	
Physician Assistant	14	23.7	45	76.3	0	0.0	
Pharm D	4	100.0	0	0.0	0	0.0	
**Facility Type**							< 0.001
Community Health	82	29.6	192	69.3	3	1.1	
Public Health	6	26.1	14	60.9	3	13.0	
Private Practice Clinic	19	15.1	107	84.9	0	0.0	
Specialist Clinic	14	58.3	10	41.7	0	0.0	
Government/Federal Agency	9	36.0	16	64.0	0	0.0	
Managed Care Health Clinic	6	17.1	29	82.9	0	0.0	
Other/multiple sites	16	53.3	14	46.7	0	0.0	
**Region of WA State**							0.311
East	11	24.4	33	73.3	1	2.2	
North	11	32.4	23	67.7	0	0.0	
North central	7	38.9	11	61.1	0	0.0	
Northwest	11	35.5	20	64.5	0	0.0	
Puget Sound	90	28.3	226	71.1	2	0.6	
South Central	9	26.5	23	67.7	2	5.9	
Southwest	6	26.1	17	73.9	0	0.0	
West	8	20.0	30	75.0	2	5.0	

^a^Three providers missing treatment data

### Implementation barriers

Barriers to screening and treatment are reported in Table 4. Among providers that report screening for HCV, the majority (65%) did not report any screening barriers. Providers that did report barriers cited the most common barriers being challenges with billing (10%), lack of onsite laboratory services (8%), and patient resistance or follow through with screening (6%). Among providers that reported not screening for HCV, the most common barriers were related to lack of organizational support, such as no electronic medical record reminder (23%), limited appointment times (23%), inadequate laboratory services (15%), and lack of staff (12%).

**Table 4:** Screening and treating barriers^a^

**Barriers Reported by Providers who Screen** (*N* = 521)	**n**	**%**
None	340	65.3%
Challenges with billing	54	10.4%
Lack of onsite laboratory	42	8.1%
Patient resistance/choice	31	6.0%
Lack of phlebotomy services	25	4.8%
Lack of staff to provide services	23	4.4%
EMR education	21	4.0%
Cost to patients	11	2.1%
Lack of lab courier services	7	1.3%
Reimbursement rates are too low/not cost effective	8	1.5%
Provider time	6	1.2%
Lack of supplies	4	0.8%
**Barriers Reported by Providers who Do Not Screen** (*N* = 26)		
No organizational support	6	23.1%
Too busy/not enough time	6	23.1%
Lack of phlebotomy services	4	15.4%
Lack of onsite laboratory	4	15.4%
Lack of staff to provide services	3	11.5%
Only Treat	3	11.5%
Challenges with billing	2	7.7%
Lack of lab courier services	1	3.8%
**Barriers Reported by Providers who Treat** (*N* = 153)		
None	73	47.7%
Lack of clinicians who treat Hepatitis C	27	17.6%
Patient barriers	23	15.0%
Challenges with billing	20	13.1%
Lack of staff to provide services	15	9.8%
Reimbursement rates are too low/ not cost effective	9	5.9%
Unable to accept new patients/no available follow-up appointments	6	3.9%
Need for education	3	2.0%
Lack of lab resources	1	0.7%
**Barriers Reported by Providers who Do Not Treat** (*N* = 410)		
I do not feel I have adequate knowledge to treat Hepatitis C	249	63.7%
Too busy/not enough time	78	19.9%
No organizational support	53	13.6%
I did not realize I could treat Hepatitis C	49	12.5%
Inadequate staff support	40	10.2%
I do not feel it should be part of my job	35	9.0%
No clinic protocol to treat Hepatitis C	7	1.8%
Reimbursement rates are too low/not cost effective	3	0.8%

^a^Providers could select multiple responses

Of the 153 participants that reported treating HCV, 73 (48%) identified no barriers to treatment, but those that did report barriers identified lack of clinicians who treat HCV (18%), patient barriers such as transportation, housing, and adherence (15%), challenges with billing and reimbursement (13%), and lack of staff (10%). In those providers that reported not treating HCV the main barriers to treating HCV were inadequate knowledge on how to treat HCV (64%), not enough time in clinic to do so (20%), and no organizational support (14%). Among providers that are not currently treating HCV (n = 391), 52%-(203/391) were interested in providing HCV treatment in the future if they had resources to support them such as access to clinical guidelines, support from a colleague experienced at treating HCV, and pharmacist support.

### Observed stigma, provider knowledge, and treatment of individuals with substance use disorder

The majority of providers who are treating patients with HCV reported no witnessed healthcare stigma related to quality of care in those with HCV (123/153) (Table 5). Specifically, 5% reported witnessing unwillingness to care for a person with HCV one or more times, 8% reported observing provision of poorer quality of care; and 7% witnessed colleagues talking badly about persons with HCV at least once There were no regional or facility features that were associated with these factors.

**Table 5:** Stigma, provider knowledge, and substance use co-treatment among providers treating HCV (*N* = 153)

Stigma Observed by Providers	n	%
Healthcare workers unwilling to care for a person with HCV	7	5%
Provision of poorer quality of care	12	8%
Colleagues talking badly about persons with HCV	11	7%
**Provider Knowledge**		
* Topics most uncomfortable discussing*		
Insurance coverage of HCV medications and support	81	57%
Medication drug interactions	74	50%
Treatment safety and side effects	49	33%
* Topics most comfortable discussing*		
HCV testing	147	99%
HCV treatment efficacy	124	84%
Liver fibrosis testing	122	82%
* Supports and Resources used most*		
Colleagues with experience	102	67%
Pharmacists	94	61%
Clinical Guidelines	81	53%
Online tutorials	67	44%
Project ECHO	60	39%
**Treating HCV in persons with Substance Use**		
* Willing to treat HCV in those actively using*		
Drugs	136	94%
Alcohol	134	95%
* Concerns when treating*		
Medication adherence	132	92%
Patient mental health	131	91%
Patient stability	137	95%
Providers treating HCV willingness to prescribe medications for substance use disorder^s^	54	60%
Provider located in Puget Sound region	38	70%

^a^Responses from 90 providers treating HCV in persons actively using alcohol or drugs

We asked providers that were treating HCV about their comfort in discussing certain topics with their patients and what types of support and resources they desired. The topics that providers were most uncomfortable discussing were insurance coverage of HCV medications and support (57%), medication drug interactions (50%), and treatment safety and side effects (33%). Supports and resources that were most useful in helping guide screening and treating of HCV included colleagues (67%), pharmacists (61%), clinical guidelines (53%), online tutorials (44%), and Project ECHO (39%), a medical education and care management program using video-conferencing technology [25].

The majority of treating providers were willing to treat HCV in those actively using drugs (94%) and alcohol (95%). Although almost all providers were willing to treat HCV in these populations, they almost all stated major concerns such as medication adherence (92%), patient mental health (91%), and stability (95%). Among providers who were treating HCV in patients actively using drugs and/or alcohol (*n* = 90), 60% (*n* = 54) stated that they are willing to prescribe medications for substance use disorders. The majority (70%, *n* = 38) of these providers were primarily located in the Puget Sound region of Washington State. (Table 5).

## Discussion

This descriptive study of barriers to HCV screening and treating in Washington State was conducted 3 years after the HCV Free Washington plan was implemented. Overall, screening practices across the state are very good, with 96% of responding providers across Washington State screening for HCV and few barriers identified. Although the majority (99%) of providers treated, or at least referred patients with HCV to other providers or settings, less than one third (28%) treated patients themselves, with many in primary care settings such as private practice, managed care, and community health referring to specialists. While fewer providers in public health reported screening and treatment practices, it was not clear if this was related to program and job description versus lack of willingness or organizational support.

One of the biggest identified barriers to HCV care that Washington State addressed early on was that of political will [10, 16]. By creating a state strategic plan, procuring fixed pricing for medication, simplifying access to medication, and eliminating treatment provider restrictions, Washington State has removed some barriers related to cost and access. However, there are still barriers to care for HCV that exist. The screening barriers that were identified most frequently were problems with billing and reimbursement, lack of onsite testing, and patient resistance to screening. In our study, only 5% of participants reported using point of care testing for screening. Point of care antibody testing utilizes a finger prick rather than phlebotomy to obtain a blood sample. The fingerstick test can be resulted within 60 min, and can be performed by anyone, as it only requires minimal training and can be used anywhere patients access care. Encouraging uptake of point of care testing would help reduce barriers to screening. [11, 26] Regarding billing and reimbursement, even with simplified access to medications this was consistently reported and a topic worthy of further investigation. One limitation of the Hep C Free plan is that it only applies to patients with Medicaid, so those patients with commercial insurance must follow the outlined policies/procedures of their insurance plan which may lead to confusion and hesitation to prescribe. While it is encouraging that providers are offering a referral for treatment, the referral process has inherent challenges such as incomplete referrals, inability to obtain timely appointments, and numerous patient barriers, that may delay or prevent timely linkage to care [27].

It is estimated that Washington State will need to treat 3,133 people annually between 2020 and 2030 to reach the HCV elimination goal [2]. In 2018, there were 7,300 people treated for HCV, demonstrating that this is an attainable goal [10]. In order to meet higher treatment needs and sustain the accomplishments to date, more providers across Washington State in primary and community health settings will be needed to treat and manage HCV. In our study, those providers treating HCV identified lack of trained clinicians to treat HCV as the most concerning barrier to care, suggesting potential saturation of services amongst current treating providers and a need to accrue and train more providers. In those providers not treating HCV the most concerning barrier identified was knowledge deficit. In our survey, half of non-treating providers identified an interest in treating if they had resources which included guidance from colleagues treating HCV, pharmacist support, and access to clinical guidelines. To ensure Washington State has a sufficient provider base to accomplish HCV elimination, more providers will require HCV treatment education and provider support [25, 28].

Globally, substance use either past or present which includes the use of opioids, amphetamines, cocaine, alcohol, and other drugs has been identified as a significant barrier to HCV screening and treatment [29]. Recent HCV clinical guidelines from the American Association for the Study of Liver Diseases and the Infectious Diseases Society of America recommend universal treatment for HCV and urged providers to screen for HCV and treat people living with HCV, regardless of substance use [11]. Our survey suggests that in Washington State substance use, including alcohol, is not a barrier to treatment, as > 94% of treating providers were willing to treat HCV in those using drugs including alcohol. However, there were moderate to high levels of concern in > 90% of the providers regarding medication adherence, patient stability, and mental health. This suggests that adherence stigma may be present more often than reported in this survey, in spite of evidence showing that those with substance use disorder achieve a sustained viral response to HCV treatment similar to those without substance use disorder [30]. In addition, there may be a need for more locally tailored supportive services across the state [31], as the majority of providers that reported willingness to provide substance use disorder medications were located in the urban Puget Sound area. This leaves some question of current practices in other regions of the state and an area for further investigation.

In our survey, stigma was reported as being observed by 5% of providers. Although this is a small proportion of respondents, enacted stigma toward individuals disproportionately impacted by HCV can have a profound impact in the wellbeing and outcomes of those with HCV. It can lead to decreased healthcare engagement and more concerningly decreased patient disclosure practices, [32, 33], a contradiction to the Hep C Free Washington plan [10]. Our small numbers did not allow for comparison of stigma across care settings or regions, though it suggests an area for further investigation to better focus provider education and destigmatizing health experiences.

This study has strengths and limitations. Strengths include significant involvement of implementing Washington State agencies and stakeholders, and access to contact information for a robust list of confirmed licensed providers in the State. Limitations include online self- report of information, which may lead to under or over reporting, misunderstanding of questions, or reporter bias. The survey distribution, allowing all registered providers in Washington State to opt in if they met criteria or opt out if they did not, may have led to self-selection or non-response sample bias. Additionally, there may have been selection bias leading to over-representation of HCV treatment providers in the sample, particularly among clinical pharmacists. However, given the aim of this study was to understand provider and system barriers to HCV screening and treatment, the majority of participants, including those who did screen for and treat HCV, still reported barriers which can be addressed to improve HCV treatment rates. Lastly, it is currently unknown exactly how many providers are treating HCV in Washington State. We estimated our survey response rate to be 24% based on the HCV treatment population denominator determined from the Medicaid managed care plans provider list, however this may be a under/overestimate as it includes providers who have treated HCV at least once in the last 24 months, some of whom may not be meaningfully providing HCV treatment and does not include providers who do not accept Medicaid.

## Conclusion

In conclusion, despite high adoption of screening and referral for HCV treatment by Washington State clinical providers, fewer providers themselves offer HCV treatment to their patients. To meet the goal of HCV elimination by 2030 in Washington State, more non-specialist providers will need to be trained and supported in the treatment and management of HCV, as well as in concurrent support for individuals with substance use disorders.

## Data Availability

The anonymized data collected are available as open data via the University of Washinton online data repository: https://datadryad.org/stash/share/nW8aSED4WuNUEONR_IZKTNFNqdIL2JMvwM41s68THS4.
